# Effectiveness of Virtual Reality-Based Training on Oral Healthcare for Disabled Elderly Persons: A Randomized Controlled Trial

**DOI:** 10.3390/jpm12020218

**Published:** 2022-02-04

**Authors:** Ai-Hua Chang, Pei-Chen Lin, Pei-Chao Lin, Yi-Ching Lin, Yuji Kabasawa, Cheng-Yu Lin, Hsiao-Ling Huang

**Affiliations:** 1 Department of Oral Hygiene, College of Dental Medicine, Kaohsiung Medical University, Kaohsiung 807, Taiwan; amberchang1104@gmail.com (A.-H.C.); peichenlin@kmu.edu.tw (P.-C.L.); 2 College of Nursing, Kaohsiung Medical University, Kaohsiung 807, Taiwan; pclin@kmu.edu.tw; 3 Center for Long-Term Care Research, Kaohsiung Medical University, Kaohsiung 807, Taiwan; 4 School of Dentistry, College of Dental Medicine, Kaohsiung Medical University, Kaohsiung 807, Taiwan; bemy1931@gmail.com; 5 Oral Care for Systemic Health Support, Faculty of Dentistry, School of Oral Health Care Sciences, Graduate School, Tokyo Medical and Dental University, Tokyo 113-8510, Japan; kabasawa.ocsh@tmd.ac.jp; 6 Department of Radio, TV & Film, Shih Hsin University, Taipei 116, Taiwan; cyou.lin@msa.hinet.net

**Keywords:** disabled, elderly, medical education, oral hygiene, virtual reality

## Abstract

(1) Background: Virtual reality (VR) technology is a widely used training tool in medical education. The present study aimed to evaluate the effectiveness of VR training of oral hygiene students on providing oral healthcare to disabled elderly persons. (2) Methods: A randomized controlled trial was conducted. In 2021, oral hygiene students were randomly assigned to a VR experimental group (EG; *n* = 11) and a control group (CG; *n* = 12). The EG received two-hour, thrice-repeated VR-based training interventions at 2-week, 4-week, and 6-week follow-ups. The CG received no VR-based interventions. Data were collected using a self-administered questionnaire before and immediately after each intervention. We performed generalized estimating equations to compare the responses. (3) Results: The EG exhibited a more significant improvement in oral care-related knowledge, attitude, self-efficacy, and intention at the 6-week follow-up than the CG. The students’ intention to assist the elderly in using interdental brushes (β = 0.91), with soft tissue cleaning (β = 0.53), and with oral desensitization (β = 0.53), and to have regular dental visits (β = 0.61) improved significantly at the 6-week follow-up. (4) Conclusions: VR training positively affected students’ knowledge, attitude, self-efficacy, and intentions on providing oral healthcare to disabled elderly persons.

## 1. Introduction

The use of virtual reality (VR) technology in higher education has been regarded as a promising development because its immersive and interactive features enable experiential learning. VR has played a key role in medical training during the COVID-19 pandemic when face-to-face training was not possible. Medical and healthcare students receive limited opportunities to practice occupational skills during their clinical placements due to a lack of supervision and relevant practice situations; VR can be used to address this problem and to train future medical professionals more effectively compared with traditional training methodologies [1]. VR simulators have proved to increase patient safety and reduce treatment risks associated with human errors in hospitals [2,3]. An randomized study found that, in medical students, using immersive and interactive VR improved their knowledge retention, increased their motivation to study, and decreased their fear of learning new things [4]. Nowadays, dental education uses VR to supplement or replace traditional clinical skills teaching in university for preclinical training in multiple disciplines [5,6,7,8]. VR training enhances students’ attitudes toward patients and the ability to recognize and resolve medical emergencies in dental education [9,10,11]. VR simulators with direct feedback and an objective evaluation function may become an important tool in the future of dental objective structured clinical examination (OSCE) [7,12]. Therefore, many studies have indicated that VR simulation has the potential to be an alternative to conventional dental training methods [13,14,15].

According to the United Nations, ~15% of the world’s population live with disabling conditions [16]. More than 46% of the elderly (age ≥ 60) have disabilities, and more than 250 million older adults worldwide have a moderate to severe disability. As of 2018, the elderly account for 14.1% of Taiwan’s total population, and Taiwan is expected to become a super-aged society by 2025 [17]. Poor oral health affects well-being and quality of life and increases the risk of general health issues [18]. Studies have demonstrated that professional oral healthcare can help reduce the prevalence of fever and the risk of respiratory infections in the elderly [19,20]. Oral hygiene students should be capable of providing professional oral healthcare to the elderly. Students should not only possess advanced oral care knowledge and skills but also pursue further learning to be effective in their practice. They should be trained in choosing appropriate oral care tools, demonstrating and teaching proper oral healthcare to patients or caregivers, and improving patient healthcare outcomes [21]. In addition to general oral health knowledge, operational steps and equipment selection are key parts of oral healthcare education. However, providing students with opportunities for independent learning and practice using traditional teaching methods in a classroom setting can be difficult.

VR has been adopted across the medical, dental, and nursing fields as a method for educating students on professional topics that require operational learning, such as emergency first aid, basic life-saving techniques, surgical skills, systematic clinical observation, and the diagnosis and treatment of periodontal diseases. The effectiveness of VR, especially in teaching operational skills, is no less than that of traditional teaching aids [22,23,24]. Studies have yet to develop and evaluate the effects of VR applications on the training of oral healthcare professionals. To the best of our knowledge, we developed the first VR system (PVIX VR, EPED Inc., Kaohsiung City, Taiwan) for training students in oral healthcare for disabled elderly persons, a high-risk population. The Cave Automatic Virtual Environment (CAVE) VR system allows users to self-practice skills related to the oral healthcare of disabled elderly persons on a step-by-step basis in a room-sized immersive working environment that combines stereoscopic displays, computer graphics, and motion-tracking technology to create a full-body sense of presence. We evaluated the effectiveness of the VR-based training on oral hygiene students’ provision of oral healthcare to disabled elderly persons.

## 2. Materials and Methods

### 2.1. Design and Participants

We conducted a randomized controlled trial (RCT). The protocol was approved by the Institutional Review Board of Kaohsiung Medical University Hospital (KMUHIRB-SV(II)-20200071). All participating students provided informed consent before participating in the study. The trial was also registered on ClinicalTrials.gov with the registration number (NCT05043909).

We recruited participants from the Department of Oral Hygiene at Kaohsiung Medical University during the 2021 spring semester. Participants who were third-year junior students were included in the present study. The participants who had not completed two medical and nursing care courses, including oral health care for community people and long-term care residents, before the study commenced were excluded.

Students were recruited according to the minimum sample size estimated based on a pilot study with an expected effect size (ES) = 2.0, a type I error = 0.05, and power = 80%. The total sample size calculated using repeated measures analysis of variance, with within–between interaction in G*Power 3.1.5 [25], was 4 in each group. In total, 11 (100%) and 12 (100%) students in the EG and CG, respectively, completed the study at all time points.

### 2.2. Randomization and Allocation

Of the students, 11 were randomized into the VR experimental group (EG) and 12 into the control group (CG) using a random table to accomplish the randomization at a ratio of 1:1. An open-label trial was used in which both the investigator and the students are aware of the VR being given. 

### 2.3. Instrument

We developed a structured questionnaire related to the oral health of the disabled elderly to collect data on five parts: (1) demographics (gender and age), (2) oral care-related knowledge, (3) attitude toward oral healthcare, (4) self-efficacy of oral healthcare, and (5) intention to assist in oral healthcare behaviors. A panel of experts reviewed the items to assess the content validity of the questionnaire; the content validity index was 0.97–1.00. The reliability of each scale was assessed in terms of internal consistency using Cronbach’s alpha. We employed a satisfaction survey based on a system usability scale (SUS) to measure the usability of the VR-based training system. The SUS addressed the effectiveness (defined as users’ ability to complete tasks using the system and the output quality of those tasks) and efficiency (the amount of resources consumed in performing tasks) of the system and the students’ satisfaction with the system (the users’ subjective reactions to using the system) [26]. We converted each student’s responses to the survey into a single score of 0–100 and assigned a rating using a curved grading scale (CGS) [27].

### 2.4. Outcome Measures

#### 2.4.1. Oral Care-Related Knowledge

Twenty statements were used to measure students’ knowledge of the oral care of the disabled elderly (e.g., “Dentures should be cleaned with toothpaste every day”) (Supplementary Table S1). The possible responses included “True” (1), “False” (0), and “I do not know” (0), with possible scores of 0–20; a higher score indicated a higher degree of oral care-related knowledge. The Kuder–Richardson 20 reliability coefficient for the scale was 0.56.

#### 2.4.2. Attitude toward Oral Healthcare

Seven statements were used to measure students’ attitudes toward oral care for the disabled elderly, such as “Oral care is as important as physical care for the elderly with disabilities.” (Supplementary Table S2). Each item was scored on a 5-point Likert-type scale ranging from 1 (“strongly disagree”) to 5 (“strongly agree”). The possible scores were 7–35, with a higher score indicating a more positive attitude toward oral care for the disabled elderly. The Cronbach’s alpha for the scale was 0.70.

#### 2.4.3. Self-Efficacy of Oral Healthcare

Eleven statements were used to evaluate students’ self-efficacy in providing the disabled elderly with oral care. Students indicated their degrees of agreement with statements related to perceptions of personal ability concerning oral care for the disabled elderly (e.g., “I am confident about assisting the elderly with disabilities in performing soft tissue cleaning”) (Supplementary Table S3). Students scored each item on a 5-point Likert-type scale ranging from 1 (“strongly disagree”) to 5 (“strongly agree”). The possible scores were 11–55, with a higher score indicating a greater degree of confidence. The Cronbach’s alpha for the scale was 0.87.

#### 2.4.4. Intention to Assist in Oral Care Behaviors

Eleven statements were used to evaluate students’ intention to assist disabled elderly persons in oral care behaviors. Students indicated their goals of promoting new oral care behaviors or modifying the existing behaviors of the disabled elderly (e.g., “I will take the initiative to check the suitability of oral care tools for the elderly with disabilities”) (Supplementary Table S4). Students scored each item on a 5-point Likert-type scale ranging from 1 (“strongly disagree”) to 5 (“strongly agree”). The possible scores were 11–55, with a higher score indicating a higher possibility of performing the behaviors. The Cronbach’s alpha for the scale was 0.92.

### 2.5. Interventions

**Figure 1:**
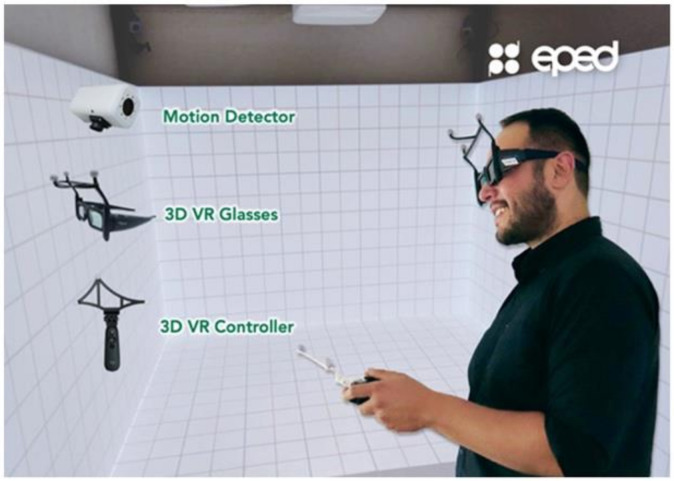
Depiction of three-dimensional (3D) virtual reality (VR) controller, glasses, and motion detectors used in the Cave Automatic Virtual Environment (CAVE) VR system during interventions.

The VR-based training system (PVIX VR, EPED Inc.) is equipped with a portable, wearable optical device that utilizes motion recognition and positioning technology and built-in virtual interactive software to assist in the self-training of future oral healthcare providers. Students are immersed in a virtual three-dimensional (3D) environment as soon as they put on the optical 3D glasses. The interactive software simulates diverse ability levels through the simulation of different actions. Students can learn standard procedures of oral healthcare, and their skills are evaluated through an automatic assessment process. In addition, the software customizes courses and lesson plans on standard processes in several areas (Figure 1).

**Figure 2:**
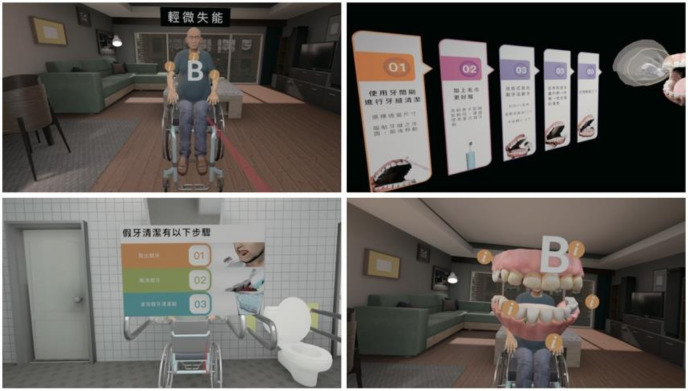
Screen captures of oral healthcare training scenarios in the interactive and immersive virtual reality (VR) system.

The learning module was divided into three sessions according to the physical conditions (mild disability, semi-disability, and total disability) and oral conditions (wearing dentures and missing teeth) of the elderly (Supplementary Table S5). Students simulated the treatment of the elderly with different physical and oral conditions using virtual situations and employed suitable oral care methods depending on each situation (Figure 2).

**Figure 3:**
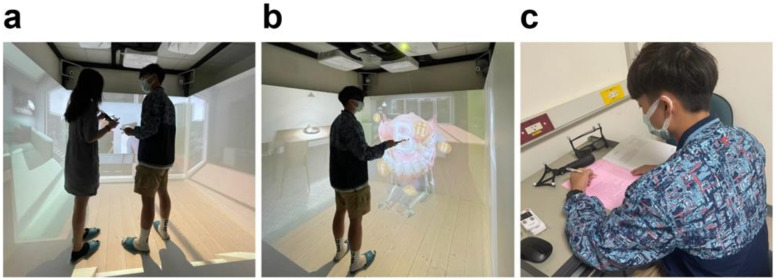
Procedure of the virtual reality (VR)-based training. (**a**) A short introduction by the researcher. (**b**) A student using the VR-based curriculum. (**c**) Evaluation.

In this study, students in the EG received thrice-repeated VR-based training for the oral healthcare of disabled elderly persons at 2-week, 4-week, and 6-week follow-ups. A research appointment was made with students for one-on-one assistance from a researcher in their free time; intervention was conducted at the VR oral care training classroom in Kaohsiung Medical University. Each training session involved two steps and took ~2 h for each student. First, the students received a short introduction to using the VR system (10 min). Second, they administered oral care to virtual elderly clients while wearing VR goggles, using handheld controllers, and listening to audio guides (90 min). After each intervention, we evaluated the students (10 min) (Figure 3). Students in the CG did not receive any VR-based interventions throughout the study.

### 2.6. Data Collection

Students in both groups self-completed a baseline (Time 1) questionnaire. We collected posttest data from the EG immediately after each intervention, and we collected the satisfaction survey at the end of the intervention period. For the CG, we collected posttest data at Time 2, Time 3, and Time 4 follow-ups.

### 2.7. Statistical Analysis

We conducted statistical analyses using STATA version 13.1 (Stata, College Station, Texas, USA). We used Fisher’s exact test and the Wilcoxon rank-sum test to compare the demographic variables of the EG and CG. The Wilcoxon signed-rank test was used to compare the oral care-related knowledge, attitude, self-efficacy, and intention to assist in oral healthcare behaviors within each group. We applied a linear regression model using generalized estimating equations (GEEs) to the comparisons of oral care-related knowledge, attitude toward oral care, self-efficacy of oral care, and intention to assist in oral care behaviors between the EG and CG. The intervention effects were adjusted for gender. We calculated the effect sizes (ESs, based on Cohen’s *d*) of the continuous variables using the mean difference in ES between the baseline and each follow-up time point for the EG and CG. An ES of 0.20 was considered small, 0.50 was medium, and 0.80 was large [28].

## 3. Results

### 3.1. Recruitment

**Figure 4:**
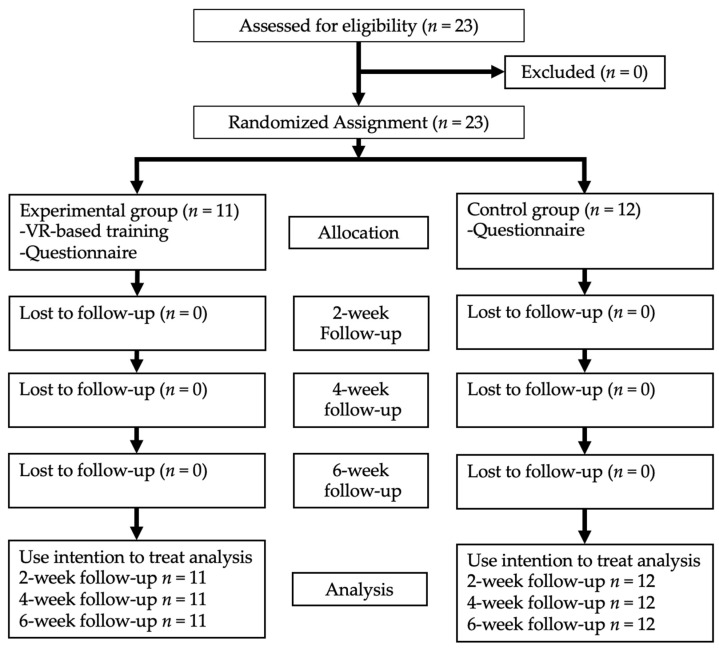
CONSORT flowchart.

Figure 4 presents the CONSORT [29] flowchart for the present randomized controlled trial. All participants completed baseline and follow-ups (Supplementary Table S6).

### 3.2. Baseline Information between the Two Groups

We observed no significant difference in sociodemographic characteristics, oral care-related knowledge, attitude toward oral healthcare, self-efficacy of oral healthcare, or intention to assist in oral care behaviors between the EG and CG at baseline (Time 1) (Table 1)**.**

### 3.3. Intervention Effects on Knowledge, Attitude, Self-Efficacy, and Intentions

The EG exhibited a greater improvement than the CG did at Times 2, 3, and 4 with respect to oral care-related knowledge, attitude toward oral healthcare, self-efficacy of oral healthcare, and intentions to assist in oral care behaviors. The ES declined from Time 1 to Time 4 in the level of knowledge (ES = 2.05, 1.23, and 1.32), attitude (ES = 0.95, 0.75, and 0.75), self-efficacy (ES = 1.75, 1.26, and 1.05), and intentions (ES=1.37, 1.35, and 0.89) (Table 2).

### 3.4. Intervention Effects on Intention to Assist in Oral Care Behaviors 

At Times 2, 3, and 4, the EG exhibited a more significant improvement than the CG did in intention to assist the elderly with disabilities in using interdental brushes (β = 0.73, ES = 1.34; β = 0.73, ES = 1.34; and β = 0.91, ES = 1.40, respectively), assist the elderly with disabilities with soft tissue cleaning (β = 0.45, ES = 0.88; β = 0.71, ES = 1.01; and β = 0.53, ES = 0.76, respectively), and remind the elderly with disabilities to have regular dental visits every 6 months (β = 0.61, ES = 1.26; β = 0.62, ES = 1.13; and β = 0.61, ES = 1.08, respectively). At Time 3, the EG exhibited a greater improvement than the CG did in intention to assist the elderly with disabilities in using the Bass brushing technique to brush their teeth (β = 0.63, ES = 0.78), to recommend proper tools for oral care to the elderly with disabilities (β = 0.53, ES = 0.96), to check the suitability of oral care tools for the elderly with disabilities (β = 0.64, ES = 1.10), and to assist the elderly with disabilities with oral desensitization before commencing an oral care session (β = 0.71, ES = 0.93). Regarding the intention to help elderly people with disabilities into a safe position before an oral care session is commenced (β = 0.62, ES = 1.13), the EG group only exhibited a better improvement than the CG did at Time 2 (Table 3).

### 3.5. SUS of the VR-Based System

The students’ mean SUS score was 75.9, corresponding to a rating of *C*. In total, 9.01% (*n* = 1), 27.27% (*n* = 3), 36.36% (*n* = 4), and 27.27% (*n* = 3) of students assigned the usability of the VR-based training system a rating of A, B, C, and D, respectively. Therefore, the PVIX system passed the usability test.

## 4. Discussion

To the best of our knowledge, this is the first study to evaluate using a VR system with an oral healthcare curriculum module to train oral hygiene students in the care of disabled elderly populations. Furthermore, we observed the highest ES in intention to assist in oral care behaviors at Time 3, but the ES value declined at Time 4. This may be interpreted as being related to learning fatigue following the thrice-repeated interventions.

Our study demonstrated that VR-based training over 6 weeks might effectively enhance students’ oral care-related knowledge, attitude, and self-efficacy. This finding is consistent with that of previous research, which indicated that immersive and interactive VR as a teaching tool could provide a more positive learning experience than traditional teaching techniques and enhance student motivation [30]. These findings are also consistent with those of another study, which demonstrated that when VR was used to explain the theoretical knowledge and skills underlying the imaging of mandibular lesions to dentistry students, the theoretical test scores and average OSCE score of the VR education group were higher than those of the traditional education group [12]. Using VR in clinical dentistry was also reported to be able to enhance students’ self-confidence [31] and improve their attitudes toward patients [9]. In other RCT studies, VR-based educational interventions have been reported to exhibit learning outcomes similar to traditional education techniques [32,33,34]. The findings from a radiography education intervention revealed that the test scores of the VR intervention group and the traditional education group both improved after training; however, the amount of change in the test scores in the VR group indicated that VR-based education may have been more effective than traditional education [35]. Clinical skill performance is reported to be the most influential source of self-efficacy [36,37]. Our findings are consistent with those of another study evaluating training outcomes for audiology students using VR or traditional training methods, which revealed that using a VR training system provided students with superior learning outcomes and self-confidence [35]. The VR training and oral healthcare curriculum employed in the present study increased students’ self-efficacy in providing oral healthcare to the elderly and educated students on appropriate oral healthcare behaviors and standard procedures, which will likely allow students to assist the elderly in preventing dental plaque formation and oral disease in the future.

In our study, we observed a greater improvement in the EG than in the CG at Time 3 follow-up in intention to assist the elderly with disabilities in using interdental brushes, using the Bass brushing technique to brush their teeth, and cleaning soft tissue; to recommend proper tools for oral care to the elderly with disabilities; to check the suitability of oral care tools for the elderly with disabilities; to remind the elderly with disabilities to have regular dental visits every 6 months; and to assist the elderly with disabilities with oral desensitization before commencing an oral care session. A previous study on neuroanatomy education revealed that integrating immersive and interactive VR into neuroanatomical training may help improve students’ knowledge and increase their intention to assist [4]. The theory of reasoned action, introduced by Fishbein in 1967 [38,39], maintains that an individual’s intention to perform a behavior determines the individual’s actual performance of that behavior. An individual’s intention to engage in healthy behaviors can predict changes in health-related behaviors. Therefore, in clinical settings, the students involved in this study may be more likely to adopt safe positioning and provide suitable oral care procedures and oral desensitization massages to the elderly while accounting for the clients’ physical and oral health conditions.

We observed large ESs for oral care-related knowledge, attitude, and self-efficacy in the EG and CG and medium-to-large ESs for intention to assist in oral care behaviors. Our new development of the CAVE VR system, which integrates immersive and interactive VR into oral healthcare, makes training reversible and repeatable by the self-practice skills on a step-by-step basis. Students can better understand the use of oral care materials and tools through the simulator’s diverse learning environment. Some studies focused on students’ acceptance of dental VR training and found that most students are willing to learn with dental simulators, which would boost their enthusiasm to learn [4,40]. The World Health Organization defines the social accountability of medical schools as the obligation to direct education, research, and service activities toward addressing the priority health concerns of the country, region, and nation that the schools have the mandate to serve [41]. Although the number of research papers on medical graduates’ preparedness for practice in certain clinical domains is rising, graduates are inadequately prepared for clinical practice [42,43]. To prepare and support medical graduates, high-quality education and training of the healthcare workforce are essential. Student preparation and skill acquisition are essential aspects in ensuring students have successful clinical placements, especially in high-acuity areas. Curriculum development should aim to assist students with their theoretical and clinical preparedness for the clinical environment. The simulation of authentic situations provided by VR has been increasingly integrated into medical education and can promote students’ acquisition of knowledge and ability to practice in a safe environment while avoiding risks and potential adverse effects on clients.

This study has several limitations. First, the small sample size may have affected the study’s outcomes. However, we observed significant effects of VR training on oral care-related knowledge, attitude, self-efficacy, and intention to assist in oral care behaviors. Second, the testing threat may have influenced the posttest results at follow-up. However, we considered the 2-week periods between the follow-ups to serve as washout time. Third, the problem of leakage from the students in the EG to those in the CG could not be avoided. Future studies can include control questions that students could not have learned from the curriculum. Finally, all participants were students in the Department of Oral Hygiene; therefore, the generalization of findings to other students should be carefully considered.

## 5. Conclusions

The use of a VR system with an oral healthcare curriculum module to train oral hygiene students in the care of disabled elderly persons impacted students’ oral care-related knowledge, attitude, self-efficacy, and intention outcomes positively at each follow-up. VR training of oral hygiene students may be adapted to better support students in their preparation for clinical practice and reduce future clinical oral care risks for the elderly with disabilities. Furthermore, the decreased ES from Time 3 to Time 4 suggests that twice-repeated VR training has a positive effect on oral hygiene students’ provision of oral healthcare to disabled elderly persons.

## Figures and Tables

**Table 1:** Baseline information of participants between two groups.

	EG (*n* = 11)		CG (*n* = 12)		
	*n*	%	*n*	%	*p*
Gender					0.466
Male	2	18.2	1	8.3	
Female	9	81.8	11	91.7	
Oral care-related knowledge, mean ± SD ^†^	18.4	±0.3	18.9	±0.3	0.224
Attitude toward oral healthcare, mean ± SD ^†^	31.0	±1.2	30.3	±0.6	0.202
Self-efficacy of oral healthcare, mean ± SD ^†^	46.0	±1.1	45.2	±1.6	0.734
Intention to assist in oral care behaviors, mean ± SD ^†^	51.6	±4.8	51.9	±5.7	0.801

^†^ Wilcoxon rank-sum test (other values are from a Fisher’s exact test); EG: experimental group; CG: control group; SD: standard deviation.

**Table 2:** Effect of change in oral care-related knowledge, attitude toward oral care, self-efficacy of oral care, and intention to assist in oral care behaviors at different stages (at baseline and at 2-week, 4-week, and 6-week follow-ups) by group among oral hygiene students.

	EG (*n* = 11)	CG (*n* = 12)	Effect Size ^d^	β	(95% CI)
	Diff ± SD ^†^	Diff ± SD^†^			
Oral Care-Related Knowledge (0–20)					
Group (EG) × Time (second)	1.3 ± 1.0	−0.3 ± 0.5	2.05	1.61	(0.92, 2.30)
Group (EG) × Time (third)	1.2 ± 1.5	−0.2 ± 0.6	1.23	1.35	(0.66, 2.04)
Group (EG) × Time (fourth)	1.3 ± 0.9	0.1 ± 0.9	1.32	1.19	(0.50, 1.88)
Effect size ^a^	1.30	0.6			
Effect size ^b^	0.80	0.33			
Effect size ^c^	1.44	0.11			
Attitude toward oral care (7–35)					
Group (EG) × Time (second)	1.5 ± 3.6	−1.3 ± 2.2	0.95	2.79	(0.56, 5.02)
Group (EG) × Time (third)	1.3 ± 3.2	−1.1 ± 3.0	0.75	2.36	(0.13, 4.59)
Group (EG) × Time (fourth)	1.7 ± 3.7	−1.2 ± 4.0	0.75	2.89	(0.66, 5.12)
Effect size ^a^	0.42	0.59			
Effect size ^b^	0.41	0.37			
Effect size ^c^	0.46	0.30			
Self-efficacy of oral care (11–55)					
Group (EG) × Time (second)	6.1 ± 3.9	−1.1 ± 4.2	1.75	7.17	(4.06, 10.29)
Group (EG) × Time (third)	6.8 ± 3.5	0.6 ± 6.0	1.26	6.23	(3.12, 9.35)
Group (EG) × Time (fourth)	5.8 ± 4.1	0.8 ± 5.3	1.05	4.98	(1.87, 8.10)
Effect size ^a^	1.56	0.26			
Effect size ^b^	1.94	0.10			
Effect size ^c^	1.41	0.15			
Intention to assist in oral care behaviors (11–55)					
Group (EG) × Time (second)	4.5 ± 5.1	−1.3 ± 3.2	1.37	5.79	(2.07, 9.51)
Group (EG) × Time (third)	6.2 ± 4.1	−0.2 ± 5.1	1.35	6.35	(2.63, 10.07)
Group (EG) × Time (fourth)	5.1 ± 5.2	0.7 ± 4.8	0.89	4.42	(0.70, 8.15)
Effect size ^a^	0.88	0.41			
Effect size ^b^	1.51	0.04			
Effect size ^c^	0.98	0.15			

Mean differences estimated adjusted by gender. Time (second): 2-week follow-up; Time (third): 4-week follow-up; Time (fourth): 6-week follow-up; EG; experimental group; CG: control group; SD: standard deviation; CI: confidence interval. ^†^ Difference between baseline and follow-up within group. ^a^ Effect size calculated as mean difference between baseline and 2-week follow-up. ^b^ Effect size calculated as mean difference between baseline and 4-week follow-up. ^c^ Effect size calculated as mean difference between baseline and 6-week follow-up. ^d^ Effect size calculated as mean difference of change between baseline and follow-up measurements between EG and CG. Reference: control group × Time 1 (baseline).

**Table 3:** Effect of change in intentions to oral care behaviors for disabled elderly persons at different stages (at baseline and at 2-week, 4-week, and 6-week follow-ups) by group among oral hygiene students.

	EG (*n* = 11)	CG (*n* = 12)		Effect Size ^d^	β	(95% CI)
	Diff ± SD ^†^	Diff ± SD ^†^				
Mouth cleaning (I will take the initiative to…)						
remind elderly people with disabilities to perform oral care after each meal						
Group (EG) × Time (second)	0.3 ± 0.6	-	-	0.61	0.27	(−0.13, 0.68)
Group (EG) × Time (third)	0.4 ± 0.5	0.2 ± 0.4		0.44	0.20	(−0.21, 0.60)
Group (EG) × Time (fourth)	0.3 ± 0.5	0.3 ± 0.6		0.04	0.02	(−0.38, 0.43)
Effect size ^a^	0.50	-				
Effect size ^b^	0.80	0.50				
Effect size ^c^	0.60	0.50				
remind elderly people with disabilities to clean their mouths before bedtime						
Group (EG) × Time (second)	0.2 ± 0.6	-	-	0.44	0.18	(−0.20, 0.56)
Group (EG) × Time (third)	0.4 ± 0.5	0.2 ± 0.4		0.44	0.20	(−0.19, 0.58)
Group (EG) × Time (fourth)	0.3 ± 0.5	0.3 ± 0.7		0.11	−0.06	(−0.44, 0.32)
Effect size ^a^	0.33	-				
Effect size ^b^	0.80	0.50				
Effect size ^c^	0.60	0.43				
assist elderly people with disabilities in cleaning their dentures						
Group (EG) × Time (second)	0.5 ± 0.7	0.2 ± 0.4		0.69	0.38	(−0.09, 0.85)
Group (EG) × Time (third)	0.5 ± 0.5	0.3 ± 0.5		0.61	0.30	(−0.18, 0.77)
Group (EG) × Time (fourth)	0.5 ± 0.8	0.4 ± 0.8		0.16	0.13	(−0.34, 0.60)
Effect size ^a^	0.71	0.50				
Effect size ^b^	1.00	0.60				
Effect size ^c^	0.63	0.50				
assist elderly people with disabilities in using the Bass brushing technique to brush their teeth						
Group (EG) × Time (second)	0.5 ± 0.7	−0.2 ± 0.6		1.27	0.71	(0.19, 1.24)
Group (EG) × Time (third)	0.5 ± 0.9	−0.1 ± 0.7		0.78	0.63	(0.11, 1.15)
Group (EG) × Time (fourth)	0.5 ± 0.8	0.1 ± 0.7		0.62	0.46	(−0.06, 0.99)
Effect size ^a^	0.71	0.33				
Effect size ^b^	0.56	0.14				
Effect size ^c^	0.63	0.14				
assist elderly people with disabilities in using the interdental brush						
Group (EG) × Time (second)	0.7 ± 0.6	0.0 ± 0.4		1.34	0.73	(0.26, 1.19)
Group (EG) × Time (third)	0.7 ± 0.8	0.0 ± 0.6		1.04	0.73	(0.26, 1.19)
Group (EG) × Time (fourth)	0.9 ± 0.5	0.0 ± 0.7		1.40	0.91	(0.44, 1.37)
Effect size ^a^	1.17	0.00				
Effect size ^b^	0.88	0.00				
Effect size ^c^	1.80	0.00				
assist elderly people with disabilities with soft tissue cleaning						
Group (EG) × Time (second)	0.4 ± 0.5	−0.1 ± 0.5		0.88	0.45	(0.00, 0.89)
Group (EG) × Time (third)	0.5 ± 0.7	−0.2 ± 0.7		1.01	0.71	(0.27, 1.16)
Group (EG) × Time (fourth)	0.4 ± 0.8	−0.2 ± 0.6		0.76	0.53	(0.08, 0.98)
Effect size ^a^	0.80	0.20				
Effect size ^b^	0.71	0.29				
Effect size ^c^	0.50	0.33				
To choose oral care tools						
recommend proper tools for oral care to elderly people with disabilities						
Group (EG) × Time (second)	0.3 ± 0.6	−0.2 ± 0.4		0.83	0.44	(0.01, 0.87)
Group (EG) × Time (third)	0.4 ± 0.5	−0.2 ± 0.6		0.96	0.53	(0.10, 0.96)
Group (EG) × Time (fourth)	0.4 ± 0.7	0.0 ± 0.6		0.57	0.36	(−0.06, 0.79)
Effect size ^a^	0.50	0.50				
Effect size ^b^	0.80	0.33				
Effect size ^c^	0.57	0.00				
Check the suitability of oral care tools for elderly people with disabilities						
Group (EG) × Time (second)	0.5 ± 0.7	−0.3 ± 0.6		1.08	0.70	(0.24, 1.17)
Group (EG) × Time (third)	0.6 ± 0.7	0.0 ±0.7		1.00	0.64	(0.17, 1.10)
Group (EG) × Time (fourth)	0.3 ± 0.5	0.0 ± 0.6		0.50	0.27	(−0.19, 0.73)
Effect size ^a^	0.71	0.50				
Effect size ^b^	1.20	0.00				
Effect size ^c^	0.60	0.00				
Dental visit						
Remind elderly people with disabilities to have regular dental visits every 6 months						
Group (EG) × Time (second)	0.3 ± 0.5	−0.3 ± 0.5		1.26	0.61	(0.22, 0.99)
Group (EG) × Time (third)	0.5 ± 0.5	−0.2 ± 0.6		1.13	0.62	(0.23, 1.01)
Group (EG) × Time (fourth)	0.4 ± 0.5	−0.3 ± 0.6		1.08	0.61	(0.23, 1.00)
Effect size ^a^	0.60	0.60				
Effect size ^b^	1.00	0.33				
Effect size ^c^	0.80	0.50				
Implement safe position						
Help elderly people with disabilities into a safe position before an oral care session is commenced						
Group (EG) × Time (second)	0.5 ± 0.5	−0.2 ± 0.6		1.13	0.62	(0.09, 1.15)
Group (EG) × Time (third)	0.6 ± 0.7	0.2 ± 0.9		0.57	0.47	(−0.06, 1.00)
Group (EG) × Time (fourth)	0.5 ± 0.8	0.1 ± 0.7		0.62	0.46	(−0.07, 0.99)
Effect size ^a^	1.00	0.33				
Effect size ^b^	0.86	0.22				
Effect size ^c^	0.63	0.14				
Implement oral desensitization						
Assist elderly people with disabilities with oral desensitization before an oral care session is commenced						
Group (EG) × Time (second)	0.2 ± 0.6	−0.3 ± 0.6		0.70	0.43	(−0.09, 0.95)
Group (EG) × Time (third)	0.5 ± 0.7	−1.2 ± 0.8		0.93	0.71	(0.19, 1.23)
Group (EG) × Time (fourth)	0.4 ± 0.8	−0.2 ± 0.7		0.70	0.53	(0.01, 1.05)
Effect size ^a^	0.33	0.50				
Effect size ^b^	0.71	1.50				
Effect size ^c^	0.50	0.29				

Mean differences estimated adjusted by gender. Time (second): 2-week follow-up; Time (third): 4-week follow-up; Time (fourth): 6-week follow-up; EG; experimental group; CG: control group; SD: standard deviation; CI: confidence interval. ^†^ Difference between baseline and follow-up within group. ^a^ Effect size calculated as mean difference between baseline and 2-week follow-up. ^b^ Effect size calculated as mean difference between baseline and 4-week follow-up. ^c^ Effect size calculated as mean difference between baseline and 6-week follow-up. ^d^ Effect size calculated as mean difference of change between baseline and follow-up measurements between EG and CG. Reference: control group × Time 1 (baseline).

## Supplementary Materials

The following supporting information can be downloaded at: https://www.mdpi.com/article/10.3390/jpm12020218/s1, Table S1: Items of oral care-related knowledge.; Table S2: Items of attitude toward oral healthcare.; Table S3: Items of self-efficacy of oral healthcare.; Table S4: Items of intention to assist in oral care behaviors.; Table S5: VR-based training curriculum module.; Table S6: CONSORT 2010 checklist of information to include when reporting a randomized trial.
