# Supplementary material for: Effectiveness of Virtual Reality-Based Training on Oral Healthcare for Disabled Elderly Persons: A Randomized Controlled Trial
Source: J Pers Med. 2022 Feb 4;12(2):218. doi: 10.3390/jpm12020218 (PMC8879228; doi:10.3390/jpm12020218)
Supplement: Supplementary file 1 [file jpm-12-00218-s001.zip › jpm-1535856-supplementary.pdf]

Supplementary Tables.

Table S1. Items of oral care-related knowledge.

Variables	Items
Oral care-related knowledge	1. Dentures should be cleaned with toothpaste every day.
	2. Dentures can be soaked in antibacterial denture cleanser overnight.
	3. Hot water must not be used to clean dentures.
	4. You need to choose a toothbrush with a small head and soft bristles to brush your natural teeth.
	5. Interdental brushes are used to remove the dental plaque that forms between teeth.
	6. There is no need to consider the size of the interdental brush.
	7. Fluoride toothpaste for adults with a fluoride content of 1350 ppm or higher can prevent tooth decay.
	8. The Bass brushing technique involves positioning the toothbrush vertically on the teeth.
	9. Using moisturizing mouthwash can mitigate dry mouth.
	10. Elderly people require regular dental checkups every 6 months.
	11. When brushing the teeth of elderly people who are bedridden, the head of the bed must be adjusted to 45–60°.
	12. When brushing the teeth of elderly people who are bedridden, care should be taken to avoid tilting their heads upward.
	13. You can use a sponge stick instead of a toothbrush for tooth cleaning.
	14. Mouthwash containing alcohol is suitable for elderly people.
	15. The sponge stick must be wrung dry after it is soaked in moisturizing mouthwash to avoid triggering a cough.
	16. Elderly people who use nasogastric feeding tubes do not require oral care.
	17. Elderly people who are completely paralyzed should be in a supine position when their teeth are brushed.
	18. Oral desensitization massage should be performed from the distal end to the proximal end.
	19. The harder the toothbrush bristles, the more thoroughly the teeth can be cleaned.
	20. Toothbrushes need to be replaced every 3 months.

Table S2. Items of attitude toward oral healthcare.

Variables	Items
Attitude toward oral healthcare	1. Assisting elderly people with disabilities in cleaning their teeth is annoying.
	2. Oral cleansing is a part of daily body cleaning.
	3. Oral care is as important as physical care for elderly people with disabilities.
	4. I don't have enough time to assist elderly people with disabilities in cleaning their teeth.
	5. It is important to take time to assist elderly people with disabilities in cleaning their teeth.
	6. Elderly people with disabilities should be reminded that regular dental check-ups are important.
	7. It is important to remind the disabled elderly to have a regular dental visit.

Table S3. Items of self-efficacy of oral healthcare.

Variables	Items
Self-efficacy of oral healthcare	1. I am confident about assisting elderly people with disabilities in cleaning their mouths.
	2. I am confident about reminding elderly people with disabilities to clean their mouths after each meal.
	3. I am confident about reminding elderly people with disabilities to clean their mouths before bed.
	4. I am confident about administering appropriate oral care depending on the oral health of the individual.
	5. I am confident in helping elderly people brush their teeth with the Bass brushing technique.
	6. I am confident about assisting elderly people with disabilities in using the interdental brush.
	7. I am confident about assisting elderly people with disabilities in performing soft tissue cleaning.
	8. I am confident about checking the suitability of oral care instruments for elderly people with disabilities.
	9. I am confident about reminding elderly people with disabilities to have regular dental visits every 6 months.
	10. I am confident in helping elderly people with disabilities into a safe position before an oral care session is commenced.
	11. I am confident in assisting elderly people with disabilities with oral desensitization before an oral care session is commenced.

Table S4. Items of intention to assist in oral care behaviors.

Variables	Items
Intention to assist in oral care behaviors	1. I will take the initiative to remind elderly people with disabilities to perform oral care after each meal.
	2. I will take the initiative to remind elderly people with disabilities to clean their mouths before bedtime.
	3. I will take the initiative to assist elderly people with disabilities in cleaning their dentures.
	4. I will take the initiative to assist elderly people with disabilities in using the interdental brush.
	5. I will take the initiative to assist elderly people with disabilities in using the Bass brushing technique to brush their teeth.
	6. I will take the initiative to assist elderly people with disabilities with soft tissue cleaning.
	7. I will take the initiative to recommend proper tools for oral care to elderly people with disabilities.
	9. I will take the initiative to check the suitability of oral care tools for elderly people with disabilities.
	10. I will take the initiative to remind elderly people with disabilities to have regular dental visits every 6 months.
	11. I will take the initiative to help elderly people with disabilities into a safe position before an oral care session is commenced.
	12. I will take the initiative to assist elderly people with disabilities with oral desensitization before an oral care session is commenced.

Table S5. VR-based training curriculum module.

Disabilities classification	Objective	Content	Time

Mild disabilities elderly	Learning oral care procedures for mild disabilities	**Denture cleaning**, including denture and the use of denture cleaning tablets<br>**Tooth cleaning**, including the use of Bass method and interdental brush<br>**Use of moisturizing mouth rinse**<br>**Dental visit reminding**	15 min
Semi-disabilities elderly	Learning oral care procedures for semi-disabilities elderly	**Safe position**<br>**Soft tissue cleaning**<br>Denture cleaning, including denture cleaning and the use of denture cleaning tablets<br>Tooth cleaning, including the use of Bass method and interdental brush<br>Dental visit reminding	25 min
Total-disabilities elderly	Learning oral care procedures for total-disabilities elderly	**Cleaning teeth in cases of nasogastric tube**<br>Safe position<br>**Oral desensitization**<br>Soft tissue cleaning<br>Tooth cleaning, including the use of Bass method and inter-dental brush<br>Dental visit reminding	25 min

Table S6. CONSORT 2010 checklist of information to include when reporting a randomized trial*.

Section/Topic	Item No	Checklist Item	Reported on Page No
		**Title and abstract**	
	1a	Identification as a randomised trial in the title	1
	1b	Structured summary of trial design, methods, results, and conclusions (for specific guidance see CONSORT for abstracts)	1
		**Introduction**	
Background and objectives	2a	Scientific background and explanation of rationale	1–2
	2b	Specific objectives or hypotheses	2
		**Methods**	
Trial design	3a	Description of trial design (such as parallel, factorial) including allocation ratio	2
	3b	Important changes to methods after trial commencement (such as eligibility criteria), with reasons	3
Participants	4a	Eligibility criteria for participants	3
	4b	Settings and locations where the data were collected	2
Interventions	5	The interventions for each group with sufficient details to allow replication, including how and when they were actually administered	4–5
Outcomes	6a	Completely defined pre-specified primary and secondary outcome measures, including how and when they were assessed	3–4
	6b	Any changes to trial outcomes after the trial commenced, with reasons	NA
Sample size	7a	How sample size was determined	3
	7b	When applicable, explanation of any interim analyses and stopping guidelines	3
Randomisation:			
Sequence generation	8a	Method used to generate the random allocation sequence	3
	8b	Type of randomisation; details of any restriction (such as blocking and block size)	3

Allocation concealment mechanism	9	Mechanism used to implement the random allocation sequence (such as sequentially numbered containers), describing any steps taken to conceal the sequence until interventions were assigned	3
Implementation	10	Who generated the random allocation sequence, who enrolled participants, and who assigned participants to interventions	3
Blinding	11a	If done, who was blinded after assignment to interventions (for example, participants, care providers, those assessing outcomes) and how	3
	11b	If relevant, description of the similarity of interventions	4–5
Statistical methods	12a	Statistical methods used to compare groups for primary and secondary outcomes	5–6
	12b	Methods for additional analyses, such as subgroup analyses and adjusted analyses	5–6
		**Results**	
Participant flow (a diagram is strongly recommended)	13a	For each group, the numbers of participants who were randomly assigned, received intended treatment, and were analysed for the primary outcome	Figure 4.
	13b	For each group, losses and exclusions after randomisation, together with reasons	6/ Figure 4.
Recruitment	14a	Dates defining the periods of recruitment and follow-up	6/ Figure 4.
	14b	Why the trial ended or was stopped	NA
Baseline data	15	A table showing baseline demographic and clinical characteristics for each group	7/ Table 1.
Numbers analysed	16	For each group, number of participants (denominator) included in each analysis and whether the analysis was by original assigned groups	6
Outcomes and estimation	17a	For each primary and secondary outcome, results for each group, and the estimated effect size and its precision (such as 95% confidence interval)	7–10
	17b	For binary outcomes, presentation of both absolute and relative effect sizes is recommended	7–10
Ancillary analyses	18	Results of any other analyses performed, including subgroup analyses and adjusted analyses, distinguishing pre-specified from exploratory	7–10
Harms	19	All important harms or unintended effects in each group (for specific guidance see CONSORT for harms)	NA
		**Discussion**	
Limitations	20	Trial limitations, addressing sources of potential bias, imprecision, and, if relevant, multiplicity of analyses	12
Generalisability	21	Generalisability (external validity, applicability) of the trial findings	12
Interpretation	22	Interpretation consistent with results, balancing benefits and harms, and considering other relevant evidence	11–12
		**Other information**	
Registration	23	Registration number and name of trial registry	2
Protocol	24	Where the full trial protocol can be accessed, if available	2
Funding	25	Sources of funding and other support (such as supply of drugs), role of funders	12–13

* We strongly recommend reading this statement in conjunction with the CONSORT 2010 Explanation and Elaboration for important clarifications on all the items. If relevant, we also recommend reading CONSORT extensions for cluster randomized trials, non-inferiority and equivalence trials, non-pharmacological treatments, herbal interventions, and pragmatic trials. Additional extensions are forthcoming: for those and for up to date references relevant to this checklist, see www.consort-statement.org
